# Proportional decline of *Anopheles quadriannulatus* and increased contribution of *An. arabiensis* to the *An. gambiae* complex following introduction of indoor residual spraying with pirimiphos-methyl: an observational, retrospective secondary analysis of pre-existing data from south-east Zambia

**DOI:** 10.1186/s13071-018-3121-0

**Published:** 2018-10-11

**Authors:** Dingani Chinula, Busiku Hamainza, Elizabeth Chizema, Deogratius R. Kavishe, Chadwick H. Sikaala, Gerry F. Killeen

**Affiliations:** 1 National Malaria Elimination Centre, Chainama Hills Hospital Grounds, PO Box 32509, Lusaka, Zambia; 2 Liverpool School of Tropical Medicine, Vector Biology Department, Pembroke Place, Liverpool, L35QA United Kingdom; 3 Ifakara Health Institute, Kiko Avenue, Environmental Health and Ecological Sciences Department, PO Box 53, Ifakara, United Republic of Tanzania

**Keywords:** *Anopheles*, Mosquito, Vector control, Indoor feeding, Outdoor feeding, Residual transmission, Monitoring, Surveillance

## Abstract

**Background:**

Across most of sub-Saharan Africa, malaria is transmitted by mosquitoes from the *Anopheles gambiae* complex, comprising seven morphologically indistinguishable but behaviourally-diverse sibling species with ecologically-distinct environmental niches. *Anopheles gambiae* and *An. arabiensis* are the mostly widely distributed major malaria vectors within the complex, while *An. quadriannulatus* is sparsely distributed.

**Methods:**

Indoor residual spraying (IRS) with the organophosphate pirimiphos-methyl (PM) was conducted four times between 2011 and 2017 in the Luangwa Valley, south-east Zambia. *Anopheles* mosquitoes were repeatedly collected indoors by several experiments with various objectives conducted in this study area from 2010 onwards. Indoor mosquito collection methods included human landing catches, Centres for Disease Control and Prevention miniature light traps and back pack aspirators. *Anopheles gambiae* complex mosquitoes were morphologically identified to species level using taxonomic keys, and to molecular level by polymerase chain reaction. These multi-study data were collated so that time trends in the species composition of this complex could be assessed.

**Results:**

The proportion of indoor *An. gambiae* complex accounted for by *An. quadriannulatus* declined from 95.1% to 69.7% following two application PM-IRS rounds with an emulsifiable concentrate formulation from 2011 to 2013, while insecticidal net utilisation remained consistently high throughout that period. This trend continued after two further rounds of PM-IRS with a longer-lasting capsule suspension formulation in 2015 and 2016/2017, following which *An. quadriannulatus* accounted for only 4.5% of the complex. During the same time interval there was a correspondingly steady rise in the proportional contribution of *An. arabiensis* to the complex, from 3.9 to 95.1%, while the contribution of nominate *An. gambiae* remained stable at ≤ 0.9%.

**Conclusion:**

It seems likely that *An. arabiensis* is not only more behaviourally resilient against IRS than *An. gambiae*, but also than *An. quadriannulatus* populations exhibiting indoor-feeding, human-feeding and nocturnal behaviours that are unusual for this species. Routine, programmatic entomological monitoring of dynamic vector population guilds will be critical to guide effective selection and deployment of vector control interventions, including supplementary measures to tackle persisting vectors of residual malaria transmission like *An. arabiensis*.

## Background

World malaria cases have fallen from 271 million in 2000 to 212 million in 2015, a reduction of 22% and within the same time period the number of deaths has equally reduced from an estimated 830,000 in 2000 to 429,000 [1]. Vector control interventions such as long-lasting insecticidal nets (LLINs) and indoor residual spraying (IRS) are responsible for most of these gains [1–3].

Malaria transmission in most parts of Africa is primarily sustained by mosquitoes from the *An. gambiae* complex and the *An. funestus* group. The *An. gambiae* complex is composed of seven cryptic sibling species, with *An. gambiae* and *An. arabiensis* being amongst the most efficient and broadly distributed malaria vectors [4–9]. The sibling species within this complex are morphologically indistinguishable, but reproductively isolated and behaviourally diverse, with ecologically distinct environmental niches distributed across sub-Saharan Africa [7, 10, 11]. For example, *An. gambiae* typically bites humans at night when they are sleeping and then rests indoors afterwards, so it is vulnerable to control with indoor interventions such as LLINs and IRS [12, 13]. *An. arabiensis* can also exhibit these same anthropophagic, endophagic behaviours, but is far more behaviourally plastic and can avoid such indoor interventions by feeding outdoors upon animals or upon people when they are unprotected [14]. Furthermore, *An. arabiensis* can also feed indoors but then rapidly escape from houses through various openings such as eaves, to rest safely outdoors without fatal exposure to insecticides [15–19]. In contrast, *An. quadriannulatus* is another sibling species from this complex that has a more limited distribution in arid areas, where it mediates little [20] or no transmission. This species is usually not considered to be a vector of malaria because it typically feeds upon animals [21, 22] which do not carry *Plasmodium* parasite species capable of infecting humans. However, in the Luangwa Valley, south-east of Zambia, it was found in sympatry with *An. gambiae* and *An. arabiensis*, where it predominantly attacked humans indoors at night in far greater numbers than the other two sibling species [22–24]. Since those original characterizations of this unusual vector guild in south-east Zambia [20], IRS with the organophosphate pirimiphos-methyl (PM) was introduced and repeated over several years [25]. Therefore, the overall goal of this retrospective observational study was to determine whether PM-IRS had any selective impact on the indoor species composition of the *An. gambiae* complex overtime.

## Methods

### Sources of retrospective data

All the studies from which the sibling species composition data are presented here, were conducted and collated from Chisobe village, situated south-east of Lusaka, the capital city of Zambia. This area is characterised by having pyrethroid-resistant *An. funestus* as the major malaria vector responsible for malaria transmission [23, 25–27]. The Zambia National Malaria Elimination Programme initiated mass LLINs distribution campaigns in 2005 and this area was one of the first beneficiaries in 2005/2006 and again in 2008/2009 [23], as well as more recently through campaigns in 2013 and 2017. Additionally, IRS has been conducted in this setting from 2011 to 2013 with an emulsifiable concentrate formulation of pirimiphos-methyl (PM) (Actellic 50EC®, Syngenta AG, Cape Town, South Africa) that has relatively short-lived effectiveness [28, 29]. This was then followed by two IRS spray rounds with a longer lasting capsule suspension formulation of PM (Actellic 300CS®, Syngenta AG, Cape Town, South Africa) [30–32] in 2015 and 2016/2017.

The species composition of the *Anopheles gambiae* complex of sibling species was evaluated as part of various studies with different objectives, conducted intermittently between 2010 and 2017. The first experiments in 2010 involved a 3 × 3 Latin Square design evaluation of three different sets of trapping methods in local houses with two blocks of three houses, with one group having LLINs alone while the other had a combination of LLINs and IRS with deltamethrine (K-Othrine WG250 Bayer Environmental Science, Johannesburg, South Africa). The first set of trapping methods consisted of human landing catches both indoors and outdoors, while the second had a CDC light trap indoors and the third had resting boxes placed indoors and outdoors. Each of the trapping methods were rotated through the three households over three consecutive nights, and this rotation schemes was repeated over 10 rounds of replication [24, 33]. In the subsequent studies in 2014, 2016 and 2017, four experimental huts of the Ifakara design [34, 35] were built in the same village and mosquitoes were collected indoors during evaluations of various IRS formulations, which were deployed as either their intended IRS format [34, 35] or treatments for window screens and eave baffles [36]. Eight adult men were recruited to sleep in the huts overnight from 19:00 to 07:00 h and the two sleepers were assigned to a single specified hut for the duration of the experiments. All the sleepers slept under an intact LLIN (Permanet.2.0, Vestergaard Frandsen, Nairobi, Kenya without any holes. Every morning at 07:00 h, indoor resting mosquitoes were retrieved using back pack aspirators [36].

In order to mitigate against sampling inconsistencies between the different collection methods used, only mosquitoes caught indoors were considered for this analysis of temporal trends in sibling species composition. All mosquitoes of the *An. gambiae* complex were initially morphologically identified to species level using various taxonomic keys [4] and recorded as unfed, partly fed, fed and gravid. Mosquitoes were then further processed to classify them to sibling species level by polymerase chain reaction (PCR) [37]. In order to avoid any introduction of any sampling bias by selective impact of insecticidal active ingredients of the LLINs, IRS or treated window screens and eave baffles, data for all PCR-amplified specimens from the *An. gambiae* complex were included, regardless of whether they were alive or dead when collected, or which collections method they were obtained with. Additionally, enzyme-linked immunosorbent assay (ELISA) was used to detect sporozoites in the heads and thoraces of the 2016 samples only.

### Data analysis

The data was analysed using the open-source statistical software package R (3.2.1) to fit generalised linear models (GLMs) with a logistic link function and binomial error distribution. Fitted GLMs assessed the effect of time (continuous predictor) on the proportion of the *An. gambiae* complex accounted for by each of the three sibling species.

## Results

**Fig. 1:**
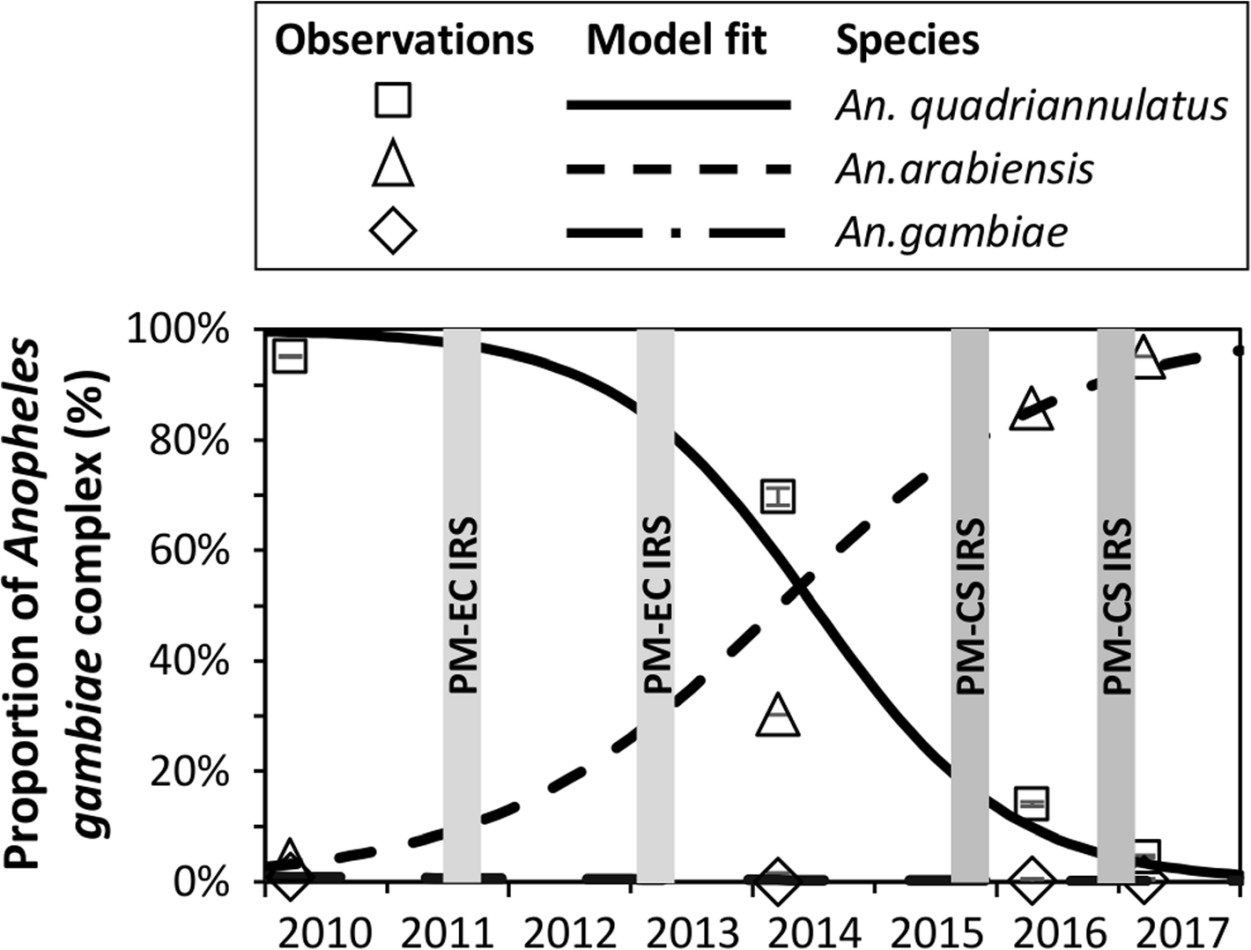
Sibling species proportional composition of the *An. gambiae* complex over time in Chisobe village. The error bars around the observations represent the 95% confidence intervals for the mean proportion accounted for by that species

From the 2010 indoor household experiments, a total of 1112 specimens of the *An. gambiae* complex were identified to species level by PCR. The proportional composition of these samples was dominated by *An. quadriannulatus* (95.2%) when compared to *An. arabiensis* (3.9%) and *An. gambiae* (0.9%). From the 2014 experiments, 218 specimens of the *An. gambiae* complex were identified to sibling species level. *Anopheles quadriannulatus* (69.7%) was still the most proportionally dominant species, but a much greater share was accounted for by *An. arabiensis* (30.3%). Out of 897 *An. gambiae* specimens identified to sibling species level in 2016, *An. arabiensis* (85.7%) was proportionally the most abundant, while *An. quadriannulatus* only accounted for a minor fraction of the complex (14.2%) and nominate *An. gambiae* remained very sparse (0.2%). In 2017, out of 285 specimens identified to species level, almost all were *An. arabiensis* (95.1%), with *An. quadriannulatus* (4.5%) and *An. gambiae* (0.4%) contributing only very minor fractions. The overall steady downward trend in the proportional contribution of *An. quadriannulatus* over the course of the study (*Z* = -21.1 *P* < 0.0001), and the corresponding upward trend for *An. arabiensis* (*Z* = -28.9, *P* < 0.0001), is presented graphically in the context of IRS rounds in Fig. 1. Only one sporozoite-positive *An. arabiensis* was identified out of 769 tested specimens from *An. gambiae* complex samples collected in 2016.

## Discussion

The steady proportional decline of indoor biting *An. quadriannulatus* and corresponding rise of *An. arabiensis* may be attributed to PM-IRS, first with a short-lived formulation in 2011 and 2013, and then followed by a more effective, longer-lasting formulation which immediately achieved even greater impact on vector densities and human malaria infection burden [25]. These observations add further evidence to support supplementing pyrethroid-treated LLINs with IRS using an alternative insecticide with a different mode of action from another chemical class [38]. This can therefore effectively control indoor-feeding secondary vectors like this local *An. quadriannulatus* population which has been incriminated previously as a vector of residual transmission in this setting [20].

On the other hand, the proportion of the *An. gambiae* complex accounted for *An. arabiensis* correspondingly increased over the same time period. It is highly unlikely that between-species differences of insecticide susceptibility could explain these observed shifts in species complex composition, because no resistance to PM has been documented to date within the *An. gambiae* complex anywhere in Zambia (NMEC, personal comms). The increased relative abundance of *An. arabiensis* may therefore have arisen from the known behavioural resilience of this species, which evades contact with insecticides by feeding on animals (zoophagic), feeding outdoors (exophagic), resting outdoors (exophilic) and expressing early-exiting behaviours that make it less vulnerable to IRS or LLINs [14, 18].

In this particular case, zoophagy seems an unlikely contributor to the preferential survival and increasing dominance of *An. arabiensis* over time, because the previously dominant species was the even more zoophagic *An. quadriannulatus*, rather than anthropophagic *An. gambiae*. Furthermore, while behavioural characterizations in this study site in 2011 and 2012 confirm that the local *An. quadriannulatus* population is indeed strongly zoophagic, these same experiments reveal an unusually strong preference for humans amongst *An. arabiensis*, which completely ignored cattle and goats when offered a choice between these three different sources of blood [20].

However, some populations of *An. arabiensis* can feed early in the evening when many people are active outdoors, where they are not protected by either LLINs or IRS. Secondly, *An. arabiensis* consistently suffer much lower mortality than either *An. gambiae* or *An. funestus* inside huts containing LLINs or IRS, even with insecticides to which they are completely susceptible [13, 39, 40], apparently because they forage far more briefly and cautiously within houses and then exit before they are fatally exposed [41–43].

While IRS clearly must have reduced *An. quadriannulatus* population densities indoors, and could have reduced inter-specific competition within the taxon sufficiently to enable genuine species replacement in the strict sense [44, 45]. However, the data presented here only represent proportions from within samples of varying size collected with varying methods on an intermittent basis, so it is not possible to unambiguously conclude that the densities of *An. arabiensis* increased in absolute rather than relative terms.

The shifting proportional balance of these two species could be readily explained by a simple reduction in the number of *An. quadriannulatus* biting indoors, even without any corresponding increase in *An. arabiensis* densities. However, it is not possible to distinguish between simple selective suppression of some species more than others [18, 46] *versus* true population replacement [44] without consistent, year-round longitudinal density measurements using fixed trapping over the full course of the period in question. Nevertheless, to the best of our knowledge this is the first report demonstrating the proportional decline of indoor-biting *An. quadriannulatus* of probably modest vectorial capacity, specifically associated with a corresponding steady rise of *An. arabiensis*, which is known to be widely important vector of residual malaria transmission across many parts of Africa [19, 42, 43]. These observations are therefore potentially important from an epidemiological and vector control perspective, especially if *An. arabiensis* has replaced *An. quadriannulatus* in the strict sense, and does prove to be a more efficient malaria vector.

As many vector control programmes re-orient themselves from malaria control to elimination, improved and novel control tools that specifically target behaviourally-evasive vectors like *An. arabiensis* should be considered as potential supplements to IRS and LLINs [47]. Emerging new approaches, such as insecticide vapour emanators, attractive toxic sugar baits and mosquito-proofed housing [48] should be prioritised for evaluation as possible ways to tackle residual malaria transmission of behaviourally-resilient vectors like *An. arabiensis.*

Of course, a retrospective observational study such as this has many limitations, notably the inconsistent and intermittent way in which mosquitoes were captured. Also, the houses and experimental huts in which they were captured varied in terms of whether their occupants used pyrethroid-treated LLINs, and whether they had been sprayed with PM. Unfortunately, it was not possible to link the molecular species identity results back to structure identity and records for the presence of LLINs or IRS treatments, so the repellent, irritant and insecticidal properties of these insecticidal measures may have affected species composition by differentially influencing the behaviour and survival of distinct sibling species. Other limitations include non-availability of data on of blood meal sources, while sporozoite rates were only assessed for the 2016 samples, with only one infected *An. arabiensis* specimen identified, so this collation of retrospective data cannot be used to infer any temporal change in sporozoite prevalence or malaria transmission. These kinds of inconsistencies are typical of such opportunistic secondary analyses of data collected with project-based research funding, because the diversity of experiments from which data were collated were originally designed to address a range of very different, loosely-related questions [18]. Also, these observations come from only one village in the Luangwa valley and cannot be considered representative of national or even provincial-level trends. Programmatically-funded and managed surveillance platforms are clearly required in Zambia and most other tropical countries for sustained, consistent longitudinal monitoring of mosquito population dynamics [49], as well as the behavioural and insecticide resistance traits that drive these trends [18, 47]. Furthermore, it will be important to quantify how much *An. arabiensis* and other persisting vectors contribute to residual malaria transmission, and to unambiguously determine whether genuine population replacement [44] of previously abundant vectors like *An. quadriannulatus* has actually occurred.

## Conclusions

Despite these study limitations, this study yields insights that further reinforces the case for establishing continuous and rigorous entomological surveillance, so as to monitor vector species abundance and composition indefinitely over time. Additionally, surveillance will be critical in order identify which vectors are contributing to residual transmission in areas with high coverage of LLINs and IRS. This will enable malaria vector control programmes to rationally deploy improved and/or novel vector control tools to specifically target vectors like *An. arabiensis* which respond poorly to control with LLINs or IRS.

## Abbreviations

CDC: Centre for disease control
HLC: Human landing catches
IRS: Indoor residual spraying
LLINs: Long lasting insecticidal nets
PM: Pirimiphos-methyl
